# Self-harm among inmates of the Berlin prison system

**DOI:** 10.3389/fpsyt.2024.1362188

**Published:** 2024-05-02

**Authors:** Alexander Blees, Sharon Jakobowitz, Jakob Hofer, Norbert Konrad, Julia Krebs, Annette Opitz-Welke

**Affiliations:** ^1^Institute of Forensic Psychiatry, Charité-Universitätsmedizin Berlin, Berlin, Germany; ^2^Department of Psychiatry and Psychotherapy, Prison Hospital Berlin, Berlin, Germany; ^3^Criminological Service for the Berlin Prison System and Social Services of the Judiciary, Berlin, Germany

**Keywords:** self-harm, mental disorder, medication, prison, prison health care

## Abstract

**Introduction:**

Self-harming behavior in prisoners is a prevalent phenomenon, with international studies estimating a 4% prevalence rate. However, studies on self-injurious behavior in the German prison system are currently lacking. Therefore, our study aims to conduct an initial assessment.

**Methods:**

The Criminological Service for the Berlin Prison System distributed questionnaires on incidents of self-harm to all Berlin prisons, except for juvenile detention centers. The questionnaires were supplemented with medical data, such as psychiatric diagnoses and medication.

**Results:**

62 questionnaires were returned, which could be attributed to 52 inmates. Compared to the average population in the Berlin prison system, the study sample exhibited variations in age, gender distribution and nationality. 94% of the inmates received a psychiatric diagnosis. Two-thirds of the male inmates had substance use disorders, while 83% of the female inmates had emotionally unstable personality resp. borderline disorders. Prior to self-harm, 87% of the inmates were administered psychiatric medication.

**Discussion:**

Our study found similarities between the study population and international studies in the distribution of certain characteristics. We assume that many of the postulated risk factors can also apply to Berlin prisoners. However, the study is limited by the small number of cases and the absence of a control group.

## Introduction

In Berlin, there were around 2,500 prisoners and those in preventive detention (excluding pre-trial detention) on the cut-off date of 03/31/2022; in Germany as a whole, there were around 42,000 prisoners (1). According to a meta-analysis by *Fazel et al.*, prisoners are more likely to suffer from mental disorders than the general population, which increases their risk of suicide and self-harm (2). The behavior of individuals not only affects those directly involved but also their immediate environment, including fellow inmates or staff (3).

According to a 2014 study of nearly 26,000 Welsh and English prisoners, the prevalence rates of self-harm were between 5-6% in male and 20-24% in female prisoners, significantly higher than the prevalence in the general population of around 1% (4). *Borrill et al.* reported a lifetime prevalence of 51% among female prisoners (5), while *Maden et al.* reported a prevalence of 17% among male prisoners (6). A more recent meta-analysis by *Favril et al.* included 35 studies conducted between 1972 and 2019, with almost 663,000 inmates (10% of whom were female), and determined a prevalence of 3.8%. The varying prevalence estimates are due to differences in survey periods, locations, non-standardized survey methods, and definitions of self-harming behavior, as opposed to suicidal behavior. Despite these differences, female prisoners consistently report higher rates of self-harming behavior (7).

The most common forms of injury among male and female prisoners were cutting and scratching (51% and 65%, respectively), followed by poisoning, overdosing or swallowing objects, strangulation and hanging (predominantly males), or choking, beating, manipulating wounds, biting and inducing hypoxia (predominantly females), with only about 1% of injuries associated with a risk of a fatal outcome (7). Although the absolute risk of completed suicide due to self-harming behavior is low, affected prisoners have a 6-8 times higher risk of suicide, which persists even after release from prison (8). Self-harming behavior can have both intrapersonal and interpersonal causes, with impaired affect regulation being the most common reason in the general population (9). Self-harm is a significant risk for inmates (10). It can also be used intentionally, such as to request a transfer to another area (11).

Although self-harming behavior is more frequent among inmates, there are only a few international studies and hardly any for Germany that examine possible risk factors or specific prevention measures. As far as can be ascertained, only one study by *Lohner & Konrad* exists for the Berlin (or German) prison system, in which 49 male prisoners with self-harming behavior were interviewed between July 2004 and July 2005. The aim was to investigate possible differences between mild and severe, potentially lethal, self-injurious behavior and possible risk factors using various psychological test instruments (12).

One of the principles of the Berlin prison law is the prevention of harmful consequences of imprisonment. International research indicates that self-harm is a prevalent occurrence in correctional facilities. However, there have been few studies on the prevalence or risk factors in Germany to date.

Our work aims to provide an initial inventory of self-harming behavior in the Berlin prison system and to narrow down and specify possible risk factors for further investigation.

Our study was based on the following hypotheses:

1. the risk factors for self-harm among prisoners described in the international literature are reflected in the frequency of certain factors in the study population (e.g. high proportion of mental illness, substance use disorders, history of self-harm, proportionally high proportion of women).
2. the individual factors prison sentence, length of previous imprisonment, number of diagnoses, number of medications, history of self-harm, history of suicidal ideation, high risk at admission (SIRAS ≥3), withdrawal symptoms in the past, nationality (natives vs. non natives), age or gender have a negative influence on the severity of self-harm (assessed with the LSARS-II).
2.1. The subgroups of inmates with violent crimes or schizophrenia show more severe self-harm than the other groups.

## Materials and methods

We distributed a questionnaire to be filled out by the responsible Social Worker in each case of self-harm, except for juvenile detention centers and youth prison. In addition to sociodemographic data, the questionnaires collected information on the prison stay and prison history (e.g., index offense, type of prison stay, sentence, disciplinary sanctions), type of self-harm, and rudimentary medical information (indications of suicidal ideations in the past, mental illness, drug use, withdrawal; collected by prison staff). The questionnaires were completed anonymously by prison staff. The questionnaire consisted of 44 questions. 12 of these questions pertained to socio-demographic and criminological data (e.g. age, gender, family status, parenthood, type of imprisonment and prison). Respondents were given the option to provide free-form or multiple-choice answers. Another item inquired about prior contact with medical, psychological, or social services, with respondents given the option to select multiple choices and provide dates. One question related to the type of self-harm (with six options available), and another inquired about previous self-harm, with respondents answering yes/no 4 items asked about possible indications of previous mental health problems, suicidal thoughts, addiction or withdrawal symptoms (yes/no, with the option to specify). 6 further questions dealt with the prison situation prior to the self-harm (e.g. special security measures, prison restrictions, accommodation), with several possible answers being offered. In addition to the objective data collected from available documentation, the prison staff was also asked 19 subjective questions about the inmate. These questions covered topics such as language communication, bullying, integration into everyday life, and coping strategies. The staff had the option to answer yes/no/or not known. We grouped these into categories for better comparability (e.g., classification of offenses as property offenses, violent offenses, etc.).

We also collected additional medical data using the BASIS-Web electronic documentation system, which is which is accessible to the medical staff of Berlin prisons. In addition, we collected the following medical data: psychiatric diagnoses during the entire period of incarceration (divided into ICD-10 categories: F1x for substance use disorders, F2x for psychotic disorders, F3x for affective disorders, F4x for stress disorders, F6x for personality disorders), psychiatrically relevant medication (divided into substance groups: Antipsychotics, Antidepressants, Mood stabilizers, Benzodiazepines, Substitutes, Opioids, Other) at the time of self-harm or up to 1 week before, and outpatient/inpatient psychiatric treatment prior to self-harm.

A total of 62 questionnaires were returned, with 8 from the women’s prison, 13 from Ploetzensee prison, and 41 from Moabit prison. The women’s prison is divided into two locations. In addition to prisoners, the women’s prisons also house juveniles and prisoners on remand. Plötzensee prison houses inmates serving alternative custodial sentences (so-called “Ersatzfreiheitstrafe”, this is imposed if a fine cannot or will not paid) and mostly short prison terms. In addition, Plötzensee Prison is affiliated with the Berlin Prison Hospital, which includes a department of psychiatry and psychotherapy. Moabit Prison is primarily used for pre-trial detention. All of the individuals examined were adults over the age of 18.

Our study serves primarily as a pilot study for the initial identification of possible risk factors for self-harm in the Berlin prison system. The baseline data are derived from the above-mentioned questionnaires that we sent to all prisons in Berlin. Due to the small number of cases (a total of 62 cases of self-harm, spread over 54 inmates), descriptive statistics are mainly calculated, and the most important socio-demographic and criminological data are compared with the surveys of the Federal Statistical Office for Berlin and the German prison system. Continuous data are presented as arithmetic mean plus standard deviation. The categorical parameters are presented as absolute frequencies and percentages. With regard to the severity of self-harm, which we determined using the LSARS-II (see below), we formed subgroups of the cases and compared them for possible differences. The continuous variables were compared using the Mann-Whitney-U-test for the variables sentence, length of imprisonment up to the time of self-harm, number of diagnoses and number of medications (no normality assumption) and an independent t-test for the variable age (normality assumption). The categorical parameters were compared using Pearson’s chi-square test and Fisher’s exact test. Due to the pilot character and the primarily descriptive approach, we did not perform a power analysis. The statistical analysis was performed using SPSS^®^ data processing software. The analyzed data is from a retrospective survey. Additionally, approval was obtained from the ethics committee of Charité University Medicine and the Criminological Service for the Berlin Prison System.

## Results

Between July 2019 and January 2021, a total of 62 cases of self-harming behaviour (53 male and 9 female cases), inolving 54 prisoners (48 male and 6 female prisoners, were evaluated. Six cases involved 2 self-injuries (5 male and 1 female cases), and 1 case involved 3 self-injuries (1 female case). In these cases, the time between the self-harm incidents ranged from 4-180 days (*M*=40.4, *SD*=57.2). It is worth noting that 2 inmates were re-imprisoned for another offense, so we treated them separately (n=54). The sociodemographic and criminological data and psychiatric diagnoses were processed on a person-related and not a case-related basis. The socio-demographic and criminological data are static values that did not change during imprisonment. The diagnoses were recorded throughout the entire period of imprisonment. It is assumed that even if a diagnosis was made after self-harm, it was already present before the event. All other data were dynamic values, i.e. the cases were evaluated here.

A total of 48 male (87%) and 6 female (11%) inmates were included. The mean age was 31.7 years with a range from 20 to 53 (*SD*=8.3) for men and 29.0 with a range from 18 to 51 (*SD*=12.0) for women. For better comparability, we created different age groups, which are shown in **Table 1**. The male detainees were of various nationalities, with German being the most frequent (15%), followed by Syrian and Polish (both 13%). Among the female detainees, German (50%), Turkish (33%) and Bosnian (16%) were most frequent. See **Table 2** for additional sociodemographic data.

**Table 1:** Age groups (in years).

	sex		total (n=54)
	male (n=48)	female (n=6)	
<21	1 (2%)	1 (17%)	2 (4%)
21-30	21 (44%)	3 (50%)	24 (44%)
31-40	18 (38%)	1 (17%)	19 (35%)
41-50	7 (15%)	0 (0%)	7 (13%)
>50	1 (2%)	1 (17%)	2 (4%)

**Table 2:** Sociodemographic data.

		sex		total (n=54)
		male (n=48)	female (n=6)	
Family status	Single	30 (63%)	5 (83%)	35 (65%)
	Married/Partnership	6 (13%)	0 (0%)	6 (11%)
	Divorced	2 (4%)	1 (17%)	3 (6%)
	Unknown	10 (21%)	0 (0%)	10 (19%)
Nationality	German	7 (15%)	3 (50%)	10 (19%)
	EU	11 (23%)	0 (0%)	11 (20%)
	Non-EU	30 (63%)	3 (50%)	33 (61%)
Parent-hood	Yes	10 (21%)	1 (17%)	11 (20%)
	No	18 (38%)	5 (83%)	23 (43%)
	Unknown	20 (42%)	0 (0%)	20 (37%)
Religion	Any confession	22 (46%)	2 (33%)	24 (44%)
	No confession	2 (4%)	1 (17%)	3 (6%)
	Unknown	24 (50%)	3 (50%)	27 (50%)

In terms of offenses, property offenses (e.g., theft, burglary, robbery) dominated among the male group at 48%, followed by violent offenses (e.g., assault, robbery with bodily injury, extortion) at 19%, and drug law violations at 13%. Similarly, women were found to be predominantly involved in property offenses, accounting for 50% of the total offenses, followed by violent offenses at 17%. Other offenses (money laundering and destruction of work equipment) accounted for 33% of the total offenses. The data shows that 33% of men were convicted with sentences between 1-87 months (*M*=21.7, *SD*=23.5), and two women were convicted, with sentences between 2-69 (*M*=31.0, *SD*=34.4). The sentences include substitute custodial sentences (enforcement of unpaid fines) and custodial sentences.

At the time of self-harm, 65% of men were remand prisoners, followed by 25% of sentenced prisoners and alternative custodial sentences with 4 In the female population, each of these categories contained 33% of cases. See **Table 3** for more criminological data.

**Table 3:** Criminological data (*one prisoner has not yet been transferred in normal imprisonment after his conviction).

		sex		total (n=54)
		male (n=48)	female (n=6)	
Offense	Property offense	23 (48%)	3 (50%)	26 (48%)
	Drug offense	6 (13%)	0 (0%)	6 (11%)
	Violence offense	9 (19%)	1 (17%)	10 (19%)
	Homicide	4 (8%)	0 (0%)	4 (7%)
	Sexual offense	4 (8%)	0 (0%)	4 (7%)
	Other	2 (4%)	2 (33%)	4 (7%)
Access Type	Freedom	37 (77%)	5 (83%)	42 (78%)
	Other prison	8 (17%)	1 (17%)	9 (17%)
	Unknown	3 (6%)	0 (0%)	3 (6%)
Conviction	Yes	16 (33%)	2 (33%)	18 (34%)
	No	30 (63%)	2 (33%)	32 (60%)
	Unknown	2 (4%)	2 (33%)	3 (6%)
Sentence lenght (months)	≤1	3 (6%)	0 (0%)	3 (6%)
	>1-2	0 (0%)	1 (17%)	1 (2%)
	>2-3	1 (2%)	0 (0%)	1 (2%)
	>3-6	2 (4%)	0 (0%)	2 (4%)
	>6-12	2 (4%)	0 (0%)	2 (4%)
	>12-59	9 (20%)	1 (17%)	10 (19%)
	≥60	1 (2%)	1 (17%)	2 (4%)
	Remand prisoner	39 (63%)	3 (50%)	33 (61%)
Type of imprisom-ent during self-harm	Remand*	31 (65%)	2 (33%)	33 (61%)
	Imprisomen	12 (25%)	2 (33%)	14 (26%)
	Alternative custodial sentence	4 (8%)	2 (33%)	6 (11%)
	Extradition custody	1 (3%)	0 (0%)	1 (2%)

Regarding the length of detention prior to self-harm, it ranged from 0-1745 days (*M*=182,2, *SD*=351.1) for males and 13-1700 days (*M*=333.78, *SD*=529.3) for females and 2-388 (*M*=195.0, *SD*=272.94). In the last 6 months prior to self-harm, 74% of male inmates were subject to security measures (special observation, placement in an two bed room) 55% because of risk to self. The figure for females was slightly higher at 89%, with, risk to self’ cited as the reason in almost all cases (89%). At the time of self-harm, 59% of the men were in solitary confinement, 21% were in a shared cell and 4% in a specially secured room. All but oneof the women were in solitary confinement (78%). Data on the course of confinement up to the time of self-harm is presented in **Table 4**.

**Table 4:** Detention history until self-harm.

		sex		total (n=62)
		male (n=53)	female (n=9)	
Days in custody	≤25	25 (47%)	1 (11%)	26 (42%)
	≤200	14 (26%)	4 (44%)	18 (29%)
	>200	14 (26%)	4 (44%)	18 (29%
Security measures during self-harm	Special observation	13 (25%)	3 (33%)	16 (26%)
	Shared room	6 (11%)	0 (0%)	6 (10%)
	Other	6 (11%)	1 (11%)	7 (11%)
	No measures	27 (51%)	5 (51%)	32 (52%)
	Unknown	1 (2%)	0 (0%)	1 (2%)
Disciplin-ary measures during self-harm	Yes	1 (2%)	0 (0%)	1 (2%)
	No	52 (98%)	9 (100%)	61 (98%)

The majority of men inflicted self-harm by cutting or stabbing (66%), followed by hanging or strangulation (9%) and swallowing objects (6%). A similar distribution is found for females with cutting and stabbing (56%), followed by hanging or strangulation and blunt force injuries (11% each). The severity of self-harm was assessed for some of the inmates using the LSARS-II, a questionnaire to assess the severity of a suicide attemp. Among the males, over 92% scored ≤3.5 and were in the low range. Only three 3 screenings were conducted for women (rated 2, 3.5, 10). Detailed data on self-harm is shown in **Table 5** and the age distribution in in **Table 6**.

**Table 5:** Self-harm data.

		sex		total
		male	female	
Type of self-harm	Cutting/Stabbing	35 (66%)	5 (56%)	40 (65%)
	Blunt force	2 (4%)	1 (11%)	3 (5%)
	Strangulating	4 (9%)	1 (11%)	6 (10%)
	Swallowing	3 (6%)	0 (0%)	3 (5%)
	Overdosing	1 (2%)	0 (0%)	1 (2%)
	Other	6 (11%)	2 (22%)	8 (13%)
	Multiple	1 (2%)	0 (0%)	1 (2%)
	**Total**	**53**	**9**	**62**
LSARS-II-Score	<3.5	44 (92%)	2 (67%)	46 (90%)
	3.5-4.5	0 (0%)	0 (0%)	0 (0%)
	≥5	4 (8%)	1 (33%)	5 (10%)
	**Total**	**48**	**3**	**52**
Self-harm in past	Yes	31 (59%)	8 (89%)	39 (63%)
	No	22 (42%)	1 (11%)	23 (37%)
	**Total**	**53**	**9**	**62**
Type of self-harm in past	Cutting/Stabbing	25 (81%)	6 (75%)	31 (80%)
	Strangulation	1 (3%)	0 (0%)	1 (3%)
	Overdosing	1 (3%)	1 (13%)	2 (5%)
	Other	4 (13%)	1 (13%)	5 (13%)
	**Total**	**31**	**8**	**39**
Suicidal ideation in past	Yes	20 (38%)	2 (22%)	22 (36%)
	No	19 (36%)	3 (33%)	22 (36%
	Unknown	14 (26%)	4 (44%)	18 (29%)
	**Total**	**53**	**9**	**62**
Suicid-score at admission	Low-risk	17 (47%)	1 (33%)	18 (46%)
	High-risk	19 (53%)	2 (67%)	21 (54%)
	**Total**	**36**	**3**	**39**

**Table 6:** Type of self-harm by age group (both sex).

		Type of self-harm						
		Cutting/stabbing	Blunt force	Strangulatin	Swallowing	Overdosing	Other	Multiple
Age group	18-20	2	0	0	0	0	0	0
	21-30	14	1	3	1	1	4	0
	31-40	12	1	1	2	0	2	1
	41-50	5	1	1	0	0	0	0
	51-60	1	0	0	0	0	1	0
Total		34	3	5	3	1	7	1

**Figure 1:**
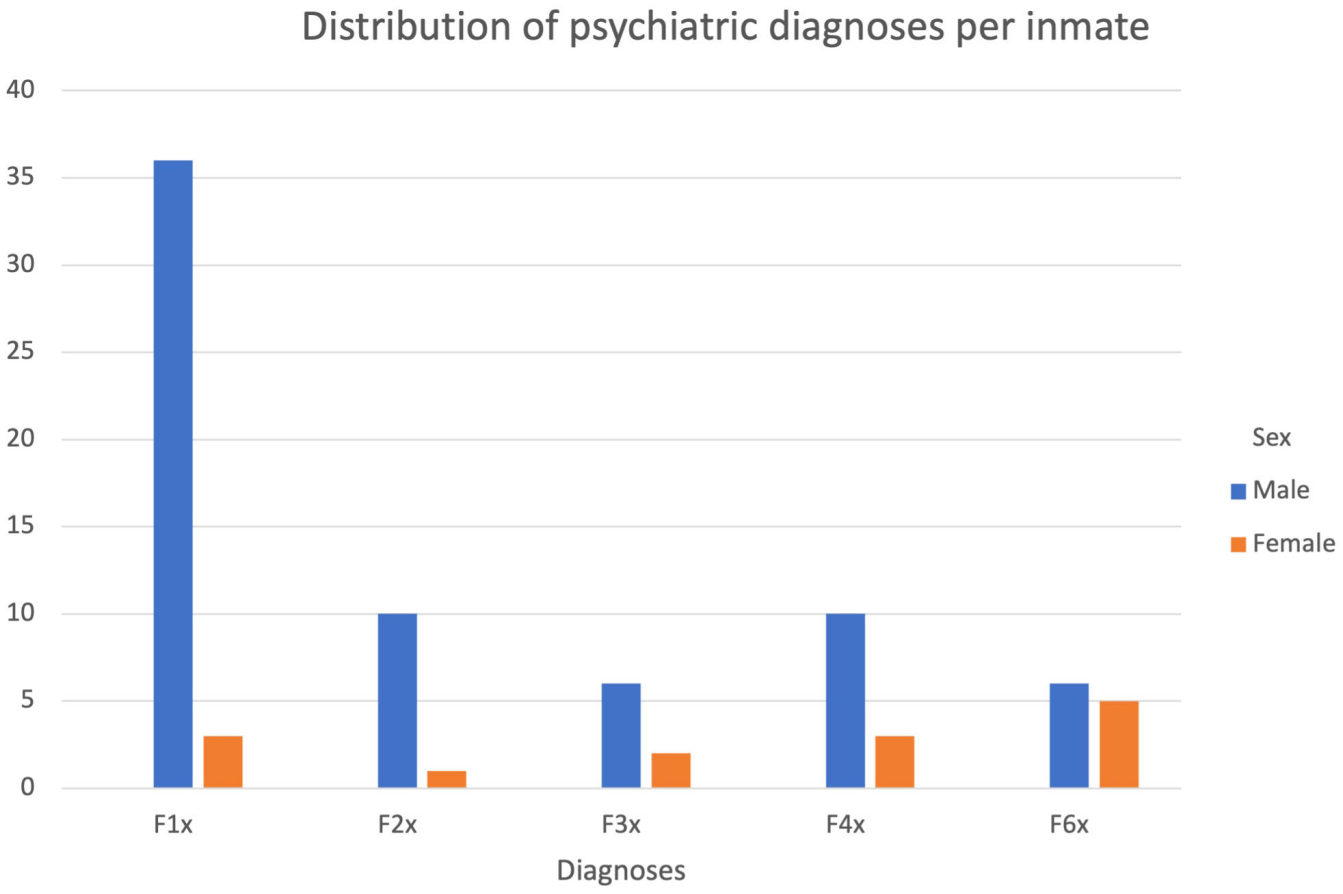
Distribution of psychiatric diagnoses. *F1x=Substance disorders, F2x=Psychotic disorders, F3x=Affective disorders, F4x=Stress disorders, F6x=Personality disorders*.

Regarding the frequency of psychiatric diagnoses, the majority of men (70%) had between one and three diagnoses. Substance use disorders were present in 80% of the sample, followed by psychotic disorders (23%) and stress disorders (21%). Among women, 5 inmates (67%) had between one and two diagnoses and the rest had four or more diagnoses. Personality disorders (83%) were among the most common diagnoses, followed by substance use disorders and stress disorders (50% each). Only 3 inmates (all men) did not have a psychiatric disorder (including substance misuse). All 3 inmates cut or stabbed themselves. Some inmates were screened for suicide on admission with the SIRAS; 53% of males and 67% of females were at risk (total ≥3). The exact distribution of diagnosis frequency and diagnoses is shown in **Table 7** and **Figure 1**. The age distribution for the type of diagnoses is shown in in **Table 8**.

**Table 7:** Number of psychiatric diagnoses and medication (up to 1 week before self-harm).

		sex		total
		male	female	
Number of diagnoses per inmate	0	3 (6%)	0 (0%)	3 (6%)
	1	11 (23%)	2 (33%)	13 (24%)
	2	12 (25%)	2 (33%)	14 (26%)
	3	11 (23%)	0 (0%)	11 (20%)
	4	6 (13%)	1 (17%)	7 (13%)
	≥5	5 (11%)	1 (17%)	6 (11%)
	**Total**	**48**	**6**	**54**
Number of psychiatric medic-ation per case	0	20 (38%)	8 (89%)	28 (45%)
	1-2	26 (49%)	0 (0%)	26 (42%)
	3-4	6 (11%)	0 (0%)	6 (10%)
	≥5	1 (2%)	1 (11%)	2 (3%)
	**Total**	**53**	**9**	**62**

**Table 8:** Diagnoses by age group (both sex), there may be several diagnoses per inmate.

		Diagnoses				
		F1x	F2x	F3x	F4x	F6x
Age group	18-20	2	0	2	0	1
	21-30	17	3	3	4	5
	31-40	13	7	5	7	4
	41-50	6	1	0	0	0
	51-60	1	0	0	2	1
Total		39	11	8	13	11

On average, men received up to six (*M*=1.1, *SD*=1.2) psychiatrically relevant medications, women up to five (*M*=0.6, *SD*=1.7). All medications received by the inmates at the time of self-harm or up to 1 week prior to self-harm were included. Among men, benzodiazepines (29%) were most commonly prescribed, followed by antipsychotics (20%) and antidepressants (18%). Among women, an antidepressant, an anticonvulsant/mood stabilizer and a substitute were each prescribed once (33% each). We also examined the frequency of outpatient and inpatient psychiatric treatment prior to self-harm. There were no outpatient or psychiatric contacts in 59% of the male cases and in 78% of the female cases. On average, there were 4.4 (*SD*=5.3) outpatient contacts for males and 4.0 (*SD*=1.41) for females. None of the male inmates had received inpatient psychiatric treatment more than once, female inmates are not treated in the psychiatric ward of the prison hospital.

**Figure 2:**
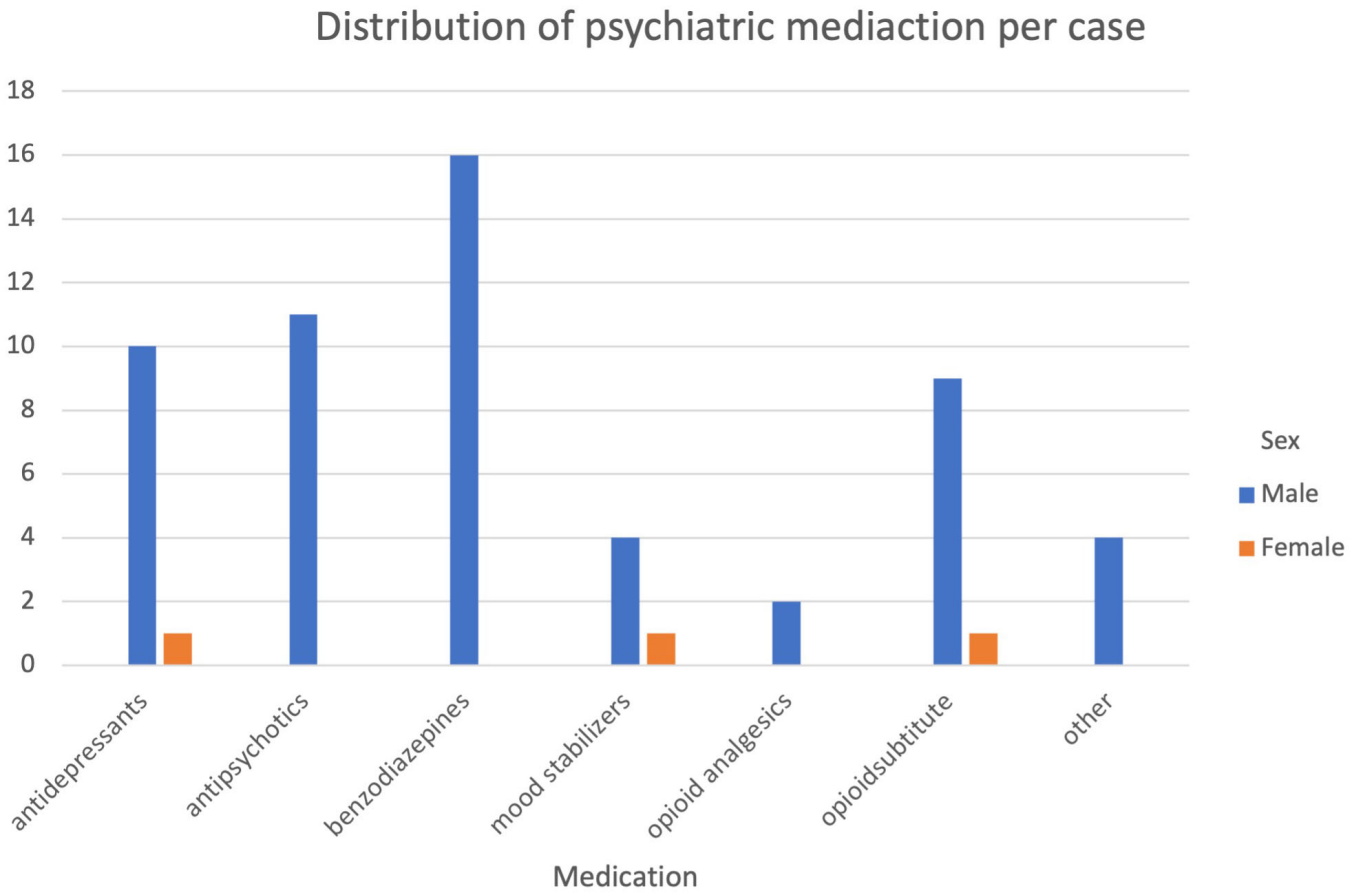
Distribution of psychotropic medications.

Further medical details are provided in **Table 9** and **Figure 2**.

**Table 9:** Medical data (*assessment by the prison staff, **only men are treated in the psychiatric ward).

		sex		total (n=62)
		male (n=51)	female (n=9)	
Outpatient psychiatric contacts before self-harm	0	31 (59%)	7 (78%)	38 (61%)
	1-3	13 (25%)	1 (11%)	14 (27%)
	4-6	2 (4%)	1 (11%)	3 (5%)
	7-9	6 (11%)	0 (0%)	6 (10%)
	>9	1 (2%)	0 (0%)	1 (2%)
Inpatient psychiatric treatment before self-harm	0	46 (87%)	9 (100%**)	55 (89%)
	1	7 (13%)	0 (0%)	7 (11%)
Whith-drawal symptoms before self-harm*	Yes	27 (51%)	1 (11%)	28 (45%)
	No	15 (28%)	6 (67%)	21 (34%)
	Unknown	11 (18%)	2 (22%)	13 (21%)

## Discussion

Self-harm among prisoners is a common phenomenon, but there is little data on it in the German prison system. Between 2019 and 2021, we asked prison staff to fill out questionnaires to provide further information on cases of self-harming behavior. We received a total of 62 questionnaires, which pertained to 54 inmates, as there were also cases of repeated self-harm.

In 11% of the cases, the inmates with self-harming behavior were women. Just under 5% of inmates in the Berlin prison system are female, which is about the German average. Looking at this ratio, an increased risk of self-harm among female prisoners in our sample can be ascertained. A similar finding can be made for the age structure. For example, 46% of our sample was under the age of 30 years; on average in Berlin, only about 26% of the prisoners (excluding remand prisoners and minors) were under 30 years of age (1). With regard to the study group, a tendency towards an increased risk of self-harm among younger prisoners can therefore be assumed. This is in line with studies of self-harm in the general population, where people who self-harm are more likely to be female (13) and younger (14), but also with studies of prisoners (4).

In Berlin, about 53% of all prisoners are German citizens. In our sample, this was the case in 18% of the sample, well below the average. The extent to which this is a possible risk factor cannot be assessed with certainty. A study from Israel found no correlation between nationality (Israeli vs. non-Israeli) (15), while an Italian study found a rate of self-harm that was twice as high among non-EU citizens (16). One possible hypothesis for the high rate of self-harm among non-German prisoners could be a language barrier and the resulting communication problems. The assessment of the prison staff that 40% of the non-German inmates did not have sufficient knowledge of German would fit in with this. A possible lack of social support should also be discussed. Only 60% of non-German citizens had German residency.

The majority of prisoners were on remand at the time of self-harm (61%). Among those with a sentence, the group with a sentence of one-five years had the highest number of self-harm cases, with 10 events (19%). This trend is consistent with figures from a study in Wales and England (4). There, 45% of self-harming prisoners were on remand, while the group sentenced to between one and four years accounted for 21%. It should be noted that in the above studies, a sentence of one-four years was associated with a lower risk of self-harm.

In 47% of our cases, the inmates were under special security measures during the period of self-harm. These are ordered in cases of imminent or existing danger to self or others and include, for example, increased observation, placement in two bed room, placement in a secure holding room or restraint. The majority of inmates were placed on these measures because they were a risk to themselves. In the last 6 months prior to self-harm, 76% of cases still had security measures in place. This may indicate that self-harm is not a singular event, but that in a larger proportion of cases, self-harm had already occurred but did not result in immediate injury or could be prevented by interventions. This is supported by the fact that 63% of cases had a history of self-harm, which is also associated with increased risk (7).

There were no discernible trends in marital status, parenthood, or criminal offenses that would indicate specific risk factors when compared to the overall Berlin and German prison populations. It is possible that the impact of marital status (and potentially parenthood) is overestimated. For instance, *Larkin et al.* found inconclusive results regarding the risk of (repeated) self-injurious behavior and marital status in general population (17). However, it is possible that the protective effect is no longer present when social structures are removed. It is important to note objectively that there was no available information on cohabitation prior to imprisonment. Regarding criminal offenses, our findings align with the literature, which also found no increased risk of certain offenses (4).

The type of self-harm reported in this study population differed only slightly from the results of the large study by *Hawton et al.* (4). In the study population, cutting and stabbing wounds were also the most common forms of self-harm, followed by object ingestion, strangulation, and blunt force trauma. The severity of self-harm was assessed using the German adaptation of the Lethality of Suicide Attemp Rating Scale-II (LSARS-II). The cut-off for a suicide attempt is ≥3.5 and for a serious suicide attempt ≥5 (18). 90% of cases had a LSARS-II score ≤3.5, indicating mild self-harm, while 10% had a score >5.0, indicating severe to life-threatening self-harm. A history of suicidal ideation was documented in 33% of inmates, which is also associated with an increased risk of self-harm (19, 20). The results of suicide screening are striking. This is done with the “Scale for Initial Risk Assessment” (SIRAS) when the inmate is admitted. This screening instrument was introduced a few years ago in the Berlin prison system; the cut-off for increased suicidality is ≥3 (21).

No significance was found looking at the relationship between the severity of self-harm (subdivided by LSARS-II score ≤3.5 and >5.0, scores between 4-5 were not available; cases n=51) and the demographic factors examined (age *p*=.49; gender *p*=.27; nationality by German, EU or non-EU *p*=.58) as well as criminological factors (sentence length by months *p*=.53; length of imprisonment until self-harm by days *p*=.11). There was also no significant predictive value to previous self-harm (*p*=.35), suicidal ideation (*p*=.22), high risk on suicide screening of ≥3 (*p*=.08), withdrawal symptoms (*p*=.70) or substance misuse (*p*>.9*)* during self-harm, between inmates with or without a psychiatric diagnosis (*p*=.73) or medication (*p*=.62), or the number of diagnoses (*p*=.61) or psychotropic medications (*p*=.57) in total to severity of self-harm. There was also no increased risk for severity of self-harm in the presence of a violent offense including sexual offense (*p*=.64) or schizophrenia (*p>*.9) (there was also no significance for any of the other subgroups).

The LSARS-II score was significantly lower in the group with documented drug misuse in the past (*p*=.04). In over 66% of cases, the injuries were cuts or stabbing wounds (not significantly different from the group with no history of drug use). Strikingly, in 66% of cases, there was evidence of withdrawal in the past. As expected, this was significantly higher (*p*<.001) than in the group with no history of drug use (although 5 withdrawals during imprisonment were also documented in this group). However, no differences were found in withdrawal symptoms during self-harm (*p=*.13*)*.

One reason for the lower LSARS-II score in the above group could therefore be an appellative purpose, such as the prescription of certain drugs or transfer to hospital, as also described by Opitz-Welke et al. (11). This would also be supported by the fact that in over 60% of cases in the group with known drug use, prison staff assumed an instrumentalized purpose of self-harm (in the group without previous drug use, the value is around 50%, *p=*.75). For further details, see **Tables 10**, **11**. A limitation is the small number of cases, which may have been even smaller due to the lack or missing of data in some subgroups (e.g. suicide screening).

**Table 10:** Severity of self-harm in group comparison (*without remand prisoners).

	LSARS-II-Score	N	Mean	SD	SEM	*p*
Age (years)	≤3.5	46	31.43	8.83	1.30	
	≥5	5	28.60	4.83	2.16	*0.49*
Sentence lenght* (months)	≤3.5	18	29.69	24.88	5.86	
	≥5	1	7	-	-	*0.53*
Imprisoment until self-harm (days)	≤3.5	46	213.63	375.49	55.36	
	≥5	5	27.00	39.73	17.77	*0.11*
Number of diagnoses	≤3.5	44	2.68	1.71	0.26	
	≥5	5	2.20	0.84	0.37	*0.61*
Number of medication	≤3.5	46	1,09	1.30	0.19	
	≥5	5	0.60	0.55	0.25	*0.57*

**Table 11:** Severity of self-harm in group comparison.

		LSARS-II Score			*p*
		≤3.5	≥5	Total	
Sex	Male	44	4	48	
	Female	2	1	3	
	Total	46	5	51	*0.27^a^*
Violence offense*	No	18	1	19	
	Yes	28	4	32	
	Total	46	5	51	*0.64^a^*
Type of imprisoment during self-harm	Remand	28	3	31	
	Imprisoment	13	1	14	
	Alternative custodial sentence	3	1	4	
	Extradition custody	2	0	2	
	Total	46	5	51	*0.71*
Type of self-harm	Cutting/Stabbing	32	3	35	
	Blunt force	2	0	2	
	Strangulationg	4	0	4	
	Swallowing	2	0	2	
	Overdosing	1	0	1	
	Other	4	2	6	
	Multiple	1	0	1	
	Total	46	5	51	*0.56*
Nationality	German	7	1	8	
	EU	10	2	12	
	Non-EU	29	2	31	
	Total	46	5	51	*0.58*
Family status	Single	30	2	32	
	Married/Partnership	5	1	6	
	Divorced	2	0	2	
	Total	37	3	40	*0.62*
Substance misuse before self-harm	No	14	4	18	
	Yes	23	0	23	
	Total	37	4	41	*0.04 ^a^*
Whithdrawal before self-harm	No	14	2	16	
	Yes	21	2	23	
	Total	35	4	39	*0.10 ^a^*
Substance misuse during self-harm	No	43	5	48	
	Yes	3	0	3	
	Total	46	5	51	>0.9^a^
Whithdrawal during self-harm	No	35	3	38	
	Yes	11	2	13	
	Total	46	5	51	*0.59 ^a^*
Suicid ideation before self-harm	No	16	2	18	
	Yes	20	0	20	
	Total	36	2	38	*0.22 ^a^*
Suicidescreening	Low-risk (<3)	15	3	18	
	High-risk (≥3)	22	0	22	
	Total	37	3	40	*0.08 ^a^*
Self-harm in the past	No	16	3	19	
	Yes	30	2	32	
	Total	46	5	51	*0.27*

*assault, homicide, total bodily injury, sexual offences; ^a^Fisher’s exact test

The high prevalence of mental disorders in the study population is remarkable. Only 6% had no psychiatric diagnosis, while the remaining inmates had at least one diagnosis. More than 80% of the men had substance misuse disorders, followed by psychotic and stress disorders. Substance misuse disorders (dependence and harmful use) were most commonly characterized by polydrug use (50%). This was followed by opioids (38%) and alcohol (32%). Five out of six female inmates had a personality disorder, and 50% were diagnosed with a substance use and stress disorder. Polydrug use and alcohol consumption dominated (67% each). Although international studies show a significantly higher prevalence of mental illness, in some cases with national differences (figures for Germany on the prevalence of mental illness are not currently available), these differed significantly from the study population in terms of frequency and distribution. In meta-analyses a prevalence of 4% for psychotic disorders (22, 23) and 47% for personality disorders is reported (23), while *Gottfried & Christopher* reported that 55-75% of U.S. prisoners report mental health problems (24). The high proportion of mental disorders in the study population may indicate an increased risk of self-harm. Several studies were able to show a significantly increased risk of psychiatric disorders and self-harm among prisoners (25, 26). With regard to the distribution of frequency of diagnosis, a link between substance use disorders and self-harm should be discussed. One possible explanation could be the occurrence of withdrawal symptoms. Prison staff reported such symptoms in 45% of cases during the period of self-harm. The proportion of personality disorders among women is also striking. Four inmates had emotionally unstable resp. borderline personality disorder and one had histrionic personality disorder. This is consistent with the findings of larger studies showing a significantly increased risk of self-harm in borderline personality disorder (25, 27, 28). In 50% of cases, inmates were receiving psychiatrically relevant medications during the week before or up to 1 week prior to self-harm, with the majority being men (58% men vs. 11% women). A possible explanation for the discrepancy between the sexes could be the distribution of diagnoses. For example, women were more likely to have personality disorders, which are difficult to treat with medication, whereas men were more likely to have psychotic disorders and substance dependence. Non-adherence to medication or a possible remission of symptoms (e.g. withdrawal symptoms) cannot be excluded. Looking at the longitudinal section, only 7 inmates (including 1 woman) were not receiving any psychiatrically relevant medication during their incarceration prior to self-harm. Looking at the international literature, there is a correlation between (past) psychiatric medication and the risk of self-harm (28, 29), so a possible risk factor can also be derived for the study population.

Prior to self-harm, 38% of cases had at least one outpatient psychiatric contact and 11% were receiving inpatient psychiatric treatment, with three cases having no psychiatric contacts prior to inpatient admission. This means that almost 44% of cases had at least one psychiatric contact of some kind prior to self-harm. It should be noted that the majority of inmates in Berlin are treated by the responsible prison doctors (mostly general practitioners or internists). Psychiatric treatment is only provided if the prison physician determines that it is necessary or if the inmate requests it. Accordingly, an above-average number of psychiatric contacts can be expected in the study population, which can be attributed to the severity of psychiatric illnesses and the resulting indication for treatment, but can also be interpreted as a possible additional risk factor for self-harm, similar to that described in previous studies (7, 30).

In summary, our study is a descriptive study of inmates with self-injurious behavior in the Berlin prison system. We were able to show that, compared to the total number of inmates in Berlin and Germany, certain characteristics identified in the international literature as risk factors for self-harm among inmates are more common in the study population. This may indicate that these risk factors also apply to a large extent to the Berlin prison population. The high proportion of psychiatric disorders in the study population is striking. In our opinion, this is a strong indication that self-injurious behavior is closely related to these disorders and represents an important risk factor, as already described in international studies. Furthermore, we performed a subgroup comparison with regard to the severity of self-harm, which we determined using the LSARS-II (we divided this into 3 categories, with only the categories ≤3.5 and ≥5.0 occurring in the study population). With the exception of a reduced risk of serious injury with a history of substance use, no significant differences were found that would indicate specific risk factors, which could be indicative of a heterogeneous distribution.

Limitations of the study include the small sample size, the lack of a control group and the fact that some of the data was collected by untrained prison staff using questionnaires. As a result, the study is merely descriptive in nature, without being able to make clear statements about possible significance or prevalence.

Self-harm in prisons is also a major problem in Germany, which poses a particular challenge to prison staff in addition to the health aspects. To date, there have been no studies in Germany that have systematically investigated this issue. With our pilot study, we wanted to identify initial risk factors and compare them with the international literature in order to get a better picture of the overall situation and identify any obvious differences. However, as prison systems vary widely internationally, this can only be an indicative study. For the future, we are planning a study with a larger number of cases and a control group to identify any specific national risk factors from which measures (e.g. screening questionnaires, similar to suicide screening) can be derived. We also plan to compare inmates with a completed suicide attempt with inmates with aggressive behavior toward others to identify possible differences.

## Data availability statement

The raw data supporting the conclusions of this article will be made available by the authors, without undue reservation.

## Ethics statement

The studies involving humans were approved by Ethikkommission der Charité – Universitätsmedizin Berlin. The studies were conducted in accordance with the local legislation and institutional requirements. Written informed consent for participation was not required from the participants or the participants’ legal guardians/next of kin in accordance with the national legislation and institutional requirements.

## Author contributions

AB: Conceptualization, Data curation, Formal analysis, Methodology, Project administration, Supervision, Validation, Visualization, Writing – original draft, Writing – review & editing. SJ: Conceptualization, Data curation, Methodology, Writing – review & editing. JH: Formal analysis, Writing – review & editing. NK: Supervision, Writing – review & editing. JK: Supervision, Writing – review & editing. AO: Conceptualization, Methodology, Supervision, Validation, Writing – review & editing.
